# Characterization of adrenal masses in young adults at a tertiary care academic medical center

**DOI:** 10.1210/jendso/bvag043

**Published:** 2026-02-26

**Authors:** Sarah Jacob, Malak Itani, Mark J Hoegger, Daniel R Ludwig, T K Pandian, Claudia Villatoro Santos, L Michael Brunt, Irina Bancos, Natalia Genere

**Affiliations:** Division of Endocrinology, Metabolism and Nutrition, Duke University Hospital, Durham, NC 27710, USA; Mallinckrodt Institute of Radiology, Washington University School of Medicine, St Louis, MO 63110, USA; Mallinckrodt Institute of Radiology, Washington University School of Medicine, St Louis, MO 63110, USA; Mallinckrodt Institute of Radiology, Washington University School of Medicine, St Louis, MO 63110, USA; Department of Surgery, Wake Forest University School of Medicine, Winston-Salem, NC 27157, USA; Division of Endocrinology, Metabolism, and Lipid Research, Department of Medicine, Washington University School of Medicine, St Louis, MO 63110, USA; Section of Minimally Invasive Surgery, Department of Surgery, Washington University School of Medicine, St Louis, MO 63110, USA; Division of Endocrinology, Diabetes, Metabolism and Nutrition, Mayo Clinic, Rochester, MN 55905, USA; Division of Endocrinology, Metabolism, and Lipid Research, Department of Medicine, Washington University School of Medicine, St Louis, MO 63110, USA; Division of Endocrinology, Diabetes, Bone and Mineral Disorders, Department of Medicine, Henry Ford Health, Detroit, MI 48202, USA

**Keywords:** adrenal mass, adrenal adenoma, epidemiology, diagnosis

## Abstract

**Context:**

Evidence on the etiology and function of adrenal masses in younger adults is scarce.

**Objective:**

This work aimed to characterize the clinical, imaging, and biochemical presentation of adrenal masses in 18- to 40-year-olds.

**Methods:**

A retrospective cohort study was conducted January 2016 to December 2021 at a tertiary academic medical center. Participants included young adults (age 18-40 years) with adrenal masses identified through radiologic registry. Exclusion criteria included preexisting adrenal masses before 2016, hyperplasia, or masses smaller than 1 cm. Outcomes included adrenal mass diagnosis, as determined by reference standard of histopathology, radiologic surveillance, and biochemical evaluation.

**Results:**

A total of 255 patients (women 142 [56%]; White 156 [61%]; median age 35 years [interquartile range 30-39 years]) were included. Comorbidities included genetic predisposition syndromes (16, 6.3%), preexisting malignancy (70, 27%), obesity (100, 48%), hypertension (89, 35%), and diabetes (25, 10%). The majority of adrenal masses were incidentally found (184, 72%) and median size was 1.9 cm (IQR 1.4-2.7 cm) on detection. Among adrenal masses in young adults, prevalence of malignancy and pheochromocytoma was 16% and 4%, respectively; among adrenal incidentalomas, 5% were malignant. Factors associated with malignancy or pheochromocytoma in multivariable analysis included nonincidental mechanism of discovery (odds ratio [OR] 15.5; 95% CI, 7.2-35.7; *P* < .001), and larger size (OR 1.5; 95% CI, 1.2-2.0; *P* < .001). Hormonal evaluation was performed in only 57 (22%) patients and was positive for hormone hypersecretion in 16 (28%) of tested individuals.

**Conclusion:**

We demonstrated a high prevalence of malignancy and pheochromocytoma in our cohort of young adults with adrenal masses. Completion of appropriate hormonal work-up was infrequent, potentially missing patients with functioning adrenal adenomas.

Over the last two decades, adrenal masses have entered the spotlight as a frequently encountered endocrine abnormality, present in about 5% to 7% of abdominal cross-sectional imaging studies [1, 2]. This statistic is driven in large part by detection of adrenal incidentalomas, which are defined as adrenal masses larger than 1 cm identified on diagnostic imaging obtained for investigations unrelated to suspected adrenal pathology or cancer evaluation [3]. The frequency of adrenal incidentalomas has increased more than 10-fold over this time, likely due to increased utilization of abdominal imaging in clinical practice, with the highest frequency of identification in those older than 65 years [4, 5].

Patients found to have an adrenal mass require further evaluation for 1) likelihood of underlying malignancy and 2) presence of hormonal hypersecretion. A general framework for evaluating and treating adrenal masses has been well described in several national and international guidelines [3, 6-8]. Risk of malignancy is primarily evaluated based on radiologic features such as mass density and size, though nonincidental mechanism of detection also increases the likelihood of an adrenal mass harboring a cancer [4, 9]. The extent of hormonal evaluation should be guided by clinical comorbidities; however, all patients benefit from completing assessment for cortisol autonomy, since 20% to 50% of adrenal tumors can harbor mild autonomous cortisol secretion [2, 5, 10, 11]. Despite these recommendations, only a small minority of adrenal masses are comprehensively evaluated with both malignancy and hypersecretion in mind [12-15].

It is unclear if current guidelines for the management of adrenal masses should be applied consistently across all age groups or if more nuanced care is required for young adults. Recent guidelines recommend urgent evaluation of patients younger than 40 years due to greater risk for malignancy or hormone excess [3]. Nevertheless, large cohort studies often leave this group underrepresented [4, 5]. Whether related to true changes in prevalence or imaging bias due to more frequent cross-sectional imaging as individuals age, the question of how age affects malignancy risk among adrenal masses is unclear. Furthermore, a recent study of patients with adrenal adenomas found that patients with Cushing syndrome (CS) and primary aldosteronism were diagnosed 5 to 10 years younger than those with mild autonomous cortisol secretion (MACS) [10]; further studies are required to better evaluate the relationship between age and likelihood of functional adrenal tumors.

In the present study, we aimed to characterize clinical, biochemical, and radiologic parameters of adrenal masses in young adults ages 18 to 40 years, and to clarify the frequency of malignancy and hormonal hypersecretion in adrenal masses in this age group.

## Materials and methods

### Study design and data source

We performed a retrospective cohort study of adrenal masses radiologically detected at Washington University School of Medicine and Barnes–Jewish Hospital in St Louis, Missouri. The study was approved by the institutional review board at Washington University. Inclusion criteria included adults ages 18 to 40 years undergoing cross-sectional abdominal imaging between January 1, 2016 and December 31, 2021. A radiology information system (RIS) database search (Montage 2022, Nuance) was conducted to identify radiology reports containing reference to an adrenal mass through use of specific search terms including “*adrenal adenoma*,” “*adrenal mass*,” “*adrenal thickening*,” “*adrenal lipid rich*,” “*indeterminate adrenal*,” as well as permutations of these terms (Supplementary Table S1 [16]). Interpreted imaging modalities were restricted to computed tomography (CT), magnetic resonance imaging (MRI), or fluorodeoxyglucose–positron emission tomography (FDG-PET). Exclusion criteria included hyperplasia without discrete nodules, patients with previously known history of adrenal masses identified on available imaging, and masses smaller than 1 cm. A formal sample size calculation was not performed; rather, the study represents a convenience sample based on available cases within the defined time frame.

### Identification of “index images”

The extracted cohort of adrenal masses was then individually reviewed to confirm the date of the “index image” for a given patient. An “index image” was defined as the first identification of the adrenal mass in a radiology report. Patients were excluded if imaging records prior to 2016 mentioned an adrenal mass. Radiology images within the inclusion time frame until December 31, 2021, were reviewed for additional radiologic characterization by other imaging modalities as described next.

### Radiologic evaluation

After appropriate studies were identified by the aforementioned protocol, 3 board-certified, subspecialized abdominal imaging radiologists (M.I., M.J.H., and D.R.L.) independently reviewed identified studies to establish imaging parameters. Twenty test studies were reviewed in consensus to confirm consistency in data collection among all radiologists.

Mass size, location, macroscopic features, attenuation by Hounsfield units (HU), and relevant adrenal imaging parameters for each modality were assessed. For adrenal protocol CT, absolute washout was defined as [early postcontrast phase HU − delayed phase HU]/[early postcontrast phase HU − noncontrast HU] × 100. Relative washout was defined as [early postcontrast phase HU − delayed phase HU]/[early postcontrast phase HU] × 100. MRI assessment included evaluation of mean signal intensity on in-phase and opposed-phase images, visual assessment of signal loss on opposed-phase images, and T2-signal intensity relative to the liver. The original radiology reports were also analyzed for descriptions of the mass in the findings (body) and impression sections of the report, radiologic diagnosis, location within report of adrenal mass characterization, and recommended follow-up if specified. For patients with multiple adrenal masses, information was collected on the largest mass only.

### Clinical evaluation

An electronic data collection form was used to collect retrospective demographic and clinical data (Research Electronic Data Capture [REDCap] 13; RRID:SCR_003445). Demographic information from the time of the index image was extracted from the electronic medical record via a data brokerage system supported through the Informatics Core Services at Washington University. Clinical history was collected within 12 months of the index image to most accurately capture disease states at the time of adrenal mass discovery. Variables included anthropometrics; vital signs; smoking status; Charlson comorbidity index [17]; presence of comorbidities including hypertension, diabetes, dyslipidemia, and osteoporosis; diagnosis and number of antihypertensive medications; and presence of any genetic predisposition syndrome.

Data were collected regarding the mechanism of detection (eg, incidental, screening for hereditary syndrome, imaging ordered for symptoms of hormonal excess, imaging ordered as part of cancer evaluation and follow-up), as well as ordering provider specialty, and whether endocrinology or endocrine surgery were involved in the evaluation. Four patients with a known genetic predisposition syndrome were found to have a new adrenal mass on imaging obtained for nonscreening or non-surveillance–related purposes, and therefore, these cases were classified as incidentals; for example, an adrenal mass was detected due during an emergency room evaluation for nephrolithiasis, so this was considered incidental detection.

Hormonal biochemical evaluation parameters were documented, including plasma renin activity (ng/mL/hour, liquid chromatography–tandem mass spectrometry (LCMS), aldosterone (ng/dL, LCMS), potassium (mmol/L), creatinine (mg/dL), post–dexamethasone cortisol (mcg/dL; Elecsys Cortisol II), adrenocorticotropin (pg/mL; Cobas electrochemiluminescence), DHEA-S (dehydroepiandrosterone sulfate; mcg/dL; Cobas electrochemiluminescence), plasma fractionated metanephrines (nmol/L, LCMS), 24-hour urinary fractionated metanephrines (mcg/24 hours, LCMS), 24-hour urinary fractionated catecholamines for epinephrine, norepinephrine, and dopamine (mcg/24 hour, LCMS), 24-hour urine cortisol (mcg/24 hours, LCMS), urine sodium (mmol/24 hour), and aldosterone (mcg/24 hour, LCMS).

Based on clinical data, the study team analyzed the presence or absence of hormone excess and deemed this complete or incomplete based on clinical and radiologic parameters. Complete hormonal evaluation was considered as at least one dexamethasone suppression test (DST) for all adrenal masses, aldosteronism evaluation in those with hypertension, and metanephrines in indeterminate or lipid-poor adrenal masses. Partial work-up was defined as completing one or more of the appropriate hormone evaluations based on history, but missing at least one hormone axis testing that was clinically indicated. No work-up defined as no hormone evaluation or only morning cortisol evaluation.

### Study outcomes

Information regarding intervention and follow-up, including biopsy, surgery, or repeat hormone evaluation, was collected. Based on review of imaging, biochemical profile, and chart review, the masses were then classified with a final diagnosis. Possible final diagnoses included the following: nonfunctioning adenoma, adenoma with unknown hormonal status, aldosteronoma, adenoma with MACS, adenoma with CS, androgen-producing adenoma, pheochromocytoma, adrenal cortical carcinoma, adrenal metastasis, other adrenal mass (eg, schwannoma, myelolipoma, hemorrhage, cyst), and indeterminate masses.

### Reference standard

The reference standard for malignancy was based on histopathology or response to therapy for primary malignancy, and additionally by biochemical abnormality in case of pheochromocytoma diagnosis. Benign adrenal masses were defined by histopathology if removed, or presence of categorically benign features on unenhanced CT; otherwise, these were considered indeterminate radiologic features. Nonfunctional categorization was assigned to masses with negative comorbidity-driven hormone evaluation. Hormonal status characterization was defined by a combination of clinical and biochemical evaluation based on recent guidelines [3]. Abnormal cortisol secretion was classified by post-DST cortisol greater than 1.8 µg/dL, elevated average midnight salivary cortisol, or elevated 24-hour urine cortisol. If biochemical evaluation was not completed, these were designated as adrenal adenoma or indeterminate adrenal mass with “unknown hormonal status.” Two authors (S.J. and N.G.) individually reviewed available data to confirm clinical diagnosis.

### Statistical analysis

All data were stored in REDCap 13 (RRID:SCR_003445) and analyzed using JMP, version 16-17 (SAS Inc; RRID:SCR_014242). We performed a descriptive summary analysis of demographic, clinical, and radiologic characteristics. Variables unavailable for part of the cohort (eg, unenhanced CT HU) were noted in table comments with specific n for number of patients in whom this variable was available. Continuous variables were summarized using mean (SD) or median (interquartile range, IQR), as appropriate based on normality of distribution. Comparisons between groups were made by *t* test or Mann-Whitney test, as appropriate. Categorical data were compared using the Pearson chi-square test or Fisher exact test, as appropriate. Individuals with missing data were excluded from analyses. Univariable and multivariable logistic regression analysis was conducted to assess independent risk factors to predict malignancy or pheochromocytoma, and included the following factors: age, sex (male), nonincidental mechanism of discovery, size at discovery (per 1-cm increase), and unenhanced HU (per 10-HU increase). Univariable analyses that had data for only a portion of the cohort (eg, unenhanced HU) were excluded from multivariable analysis. Results from models are reported as odds ratios (ORs) and 95% CIs. A probability value of *P* less than or equal to .05 was considered statistically significant for all tests.

## Results

**Figure 1:**
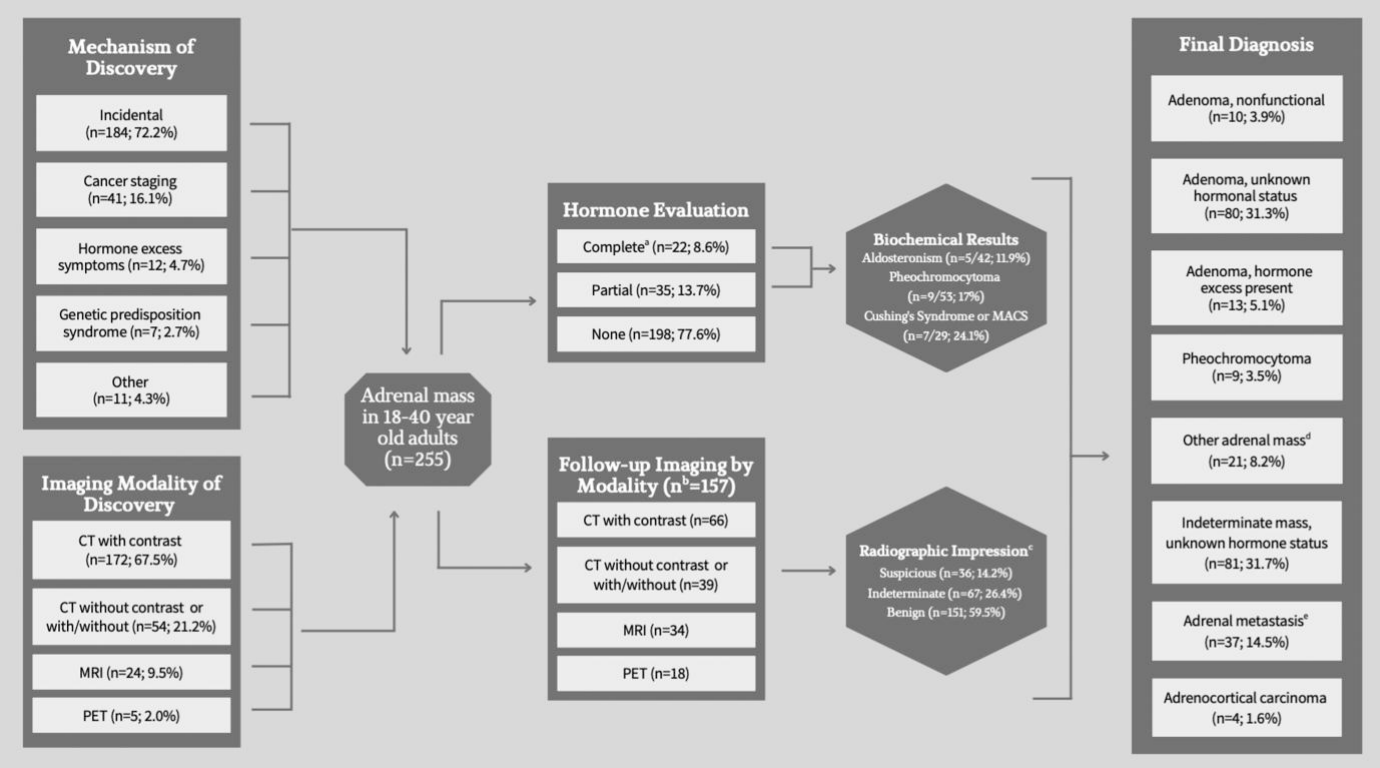
Adrenal lesion detection, evaluation, and diagnosis (n = 255). Results of clinical history, imaging, and hormone evaluation were used to categorize final diagnosis of adrenal mass. ^a^Defined as at least one dexamethasone suppression test for all adrenal masses, aldosterone and renin in those with hypertension, and metanephrines in indeterminate or lipid-poor adrenal masses. ^b^Number of unique radiographs (in all other cases, n represents individual patients). ^c^Determined based on composite of all imaging available for each mass. ^d^Group included schwannoma (n = 2), myelolipoma (n = 5), hemorrhage (n = 8), and cyst (n = 6). ^e^Most common primary malignancies included breast (7, 19%), connective tissue/musculoskeletal (5, 14%); there was equal prevalence of lymphoma, melanoma, lung carcinoma, and renal carcinomas (4, 11%).

A total of 255 adrenal masses were identified in individuals ages 18 to 40 years. Median age was 35 years (IQR 30-39 years) and the cohort included 142 women (56%), 156 White race (61%), and 86 Black race (34%) (Table 1). More than half of patients had current or prior tobacco use (124, 54%) and a minority had a hereditary predisposition syndrome such as multiple endocrine neoplasia (MEN), neurofibromatosis type 1 (NF1), von Hippel-Lindau (VHL) or familial adenomatous polyposis (FAP) (16, 6.3%). Charlson comorbidity index of the cohort was 0 (IQR 0-2), consistent with 98% 10-year survival; however, almost half were obese (body mass index [BMI] > 30, 100, 48%) and a third had hypertension (89, 35%). The majority of adrenal masses were incidentally detected (184, 72%), with nearly one-third of images being obtained from an emergency room setting; others were detected as part of cancer evaluation or follow-up (41, 16%), screening in patients with genetic predisposition syndromes (7, 2.7%), or hormonal excess evaluation (12, 5%) (Fig. 1).

**Table 1:** Baseline patient characteristics

		All adrenal masses (n = 255)	Adrenal incidentalomas*^a^* (n = 184)
Sex, female, n (%)		142 (55.7%)	99 (53.8%)
Age, median (IQR, range)*^b^*, y		35.0 (30-39, 19-41)	35.0 (31-39, 19-41)
Race, n (%)	White	156 (61.1%)	100 (54.3%)
	Black	86 (33.7%)	77 (41.8%)
	Other	13 (5.0%)	7 (3.7%)
Tobacco use, current or past, n (%)		124 (53.9%)	95 (56.9%)
Genetic predisposition syndrome, present,*^c^* n (%)		16 (6.3%)	N/A
Charlson comorbidity index, median (IQR)		0 (0-2)	0 (0-1)
Preexisting malignancy, n (%)		70 (27.4%)	27 (14.7%)
Metabolic history	BMI (median, IQR)	29.6 (25.4-37.8)	30.4 (25.8-38.4)
	BMI > 30, n (%)	100 (48.3%)	78 (52%)
	Hypertension, present, n (%)	89 (34.9%)	66 (35.9%)
	Diabetes, present, n (%)	25 (9.8%)	23 (12.5%)
	Prediabetes, present, n (%)	13 (5.1%)	10 (5.4%)
	Hyperlipidemia, present, n (%)	26 (10.2%)	17 (9.2%)
Evaluation with endocrinologist or endocrine surgeon		58/255 (22.7%)	

Continuous data are summarized as mean and SD or as median and IQRs, unless otherwise indicated. Categorical data are presented as frequencies and percentages. Data are present for all individuals unless otherwise noted in table line.

Abbreviations: BMI, body mass index; FAP, familial adenomatous polyposis; IQR, interquartile range; MEN1, multiple endocrine neoplasia 1; MEN2, multiple endocrine neoplasia 2; NF1, neurofibromatosis type 1; NF2, neurofibromatosis type 2; VHL, von Hippel-Lindau.

^*a*^Incidentaloma defined as greater than 1-cm adrenal mass detected on imaging that was obtained for evaluation of pathology unrelated to adrenal disease.

^*b*^Age rounded to nearest whole number.

^*c*^Genetic predisposition syndromes included the following: FAP (n = 6), MEN1 (n = 2), MEN2 (n = 1), NF1 (n = 2), NF2 (n = 1), and VHL (n = 4).

**Figure 2:**
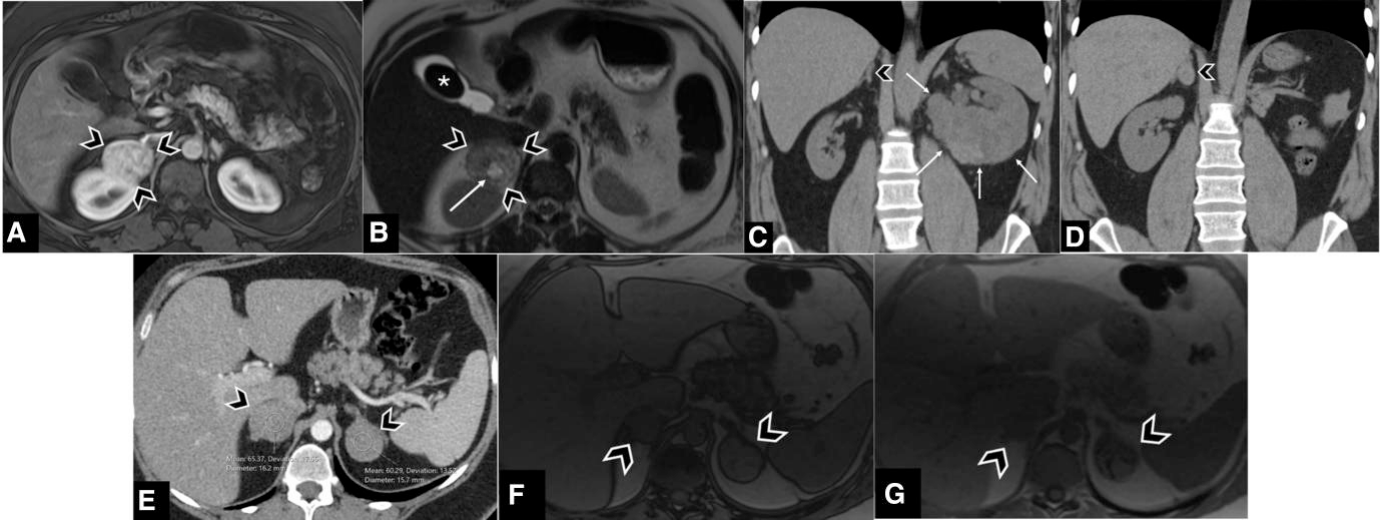
Visual guide of adrenal masses in young adults. A and B, A 46-year-old woman with neurofibromatosis type 1. Magnetic resonance imaging (MRI) demonstrates a right adrenal mass (arrowheads) with intense enhancement on A, axial T1-weighted MRI with contrast in the arterial phase, and heterogeneous signal intensity with areas of hyperintensity (white arrow) on B, T2-weighted MRI images. Pathology confirmed a pheochromocytoma. Incidentally noted is a gallstone (asterisk). C and D, A 39-year-old man presenting with hematuria, diagnosed with clear cell renal cell carcinoma (RCC) of the left kidney (white arrows in C) with a normal right adrenal gland (arrowhead in C). The patient underwent left radical nephrectomy, including left adrenalectomy. D, Computed tomography (CT) scan performed 1 year later revealed a new right adrenal nodule (arrowhead in D), consistent with RCC metastasis on biopsy, that was subsequently resected. E to G, A 32-year-old woman with MEN1 syndrome and bilateral adrenal masses (arrowheads) that demonstrate indeterminate CT density on this late arterial phase CT (right adrenal mean HU 65, left adrenal mean HU 60). There was no intralesional macroscopic nor microscopic (intravoxel) fat on MRI with no change seen in the signal intensity between F, opposed-phase and G, in-phase MRI. These lesions were indeterminate on imaging. Surgical pathology confirmed that the right adrenal mass was an oncocytic adrenal cortical neoplasm of uncertain malignant potential, and the left was consistent with adrenal cortical carcinoma.

At time of initial detection, median size was 1.9 cm (IQR 1.4-2.7 cm) and masses were bilateral in 19 (7.5%) (Table 2). Index images were most commonly CT with contrast (172, 68%). Among index images, radiology reports mentioned adrenal masses about three-quarters of the time (195, 76%), and recommendations for follow-up imaging (99, 39%) or hormonal evaluation (7, 2.7%) were less frequent. About two-thirds (157, 62%) of patients had postindex imaging scans, which were most commonly abdominal CT with contrast (66, 42%). Among all noncontrasted CT images, prevalence of lipid-rich masses (HU <10; 40/79, 51%) and lipid-poor masses (HU ≥ 10; 39/79, 49%) were similar. Among the entire cohort of adrenal masses, CT with adrenal washout protocol was used in 31 individuals (12.2%) and chemical shift MRI in 57 individuals (21.2%; see Table 2). Fig. 2 illustrates 3 representative cases of adrenal masses in young adults collected as part of the study.

**Table 2:** Adrenal mass radiologic characteristics (n = 255)

		All adrenal masses (n = 255)	Adrenal incidentalomas*^a^* (n = 184)
Size, median (IQR, range), cm		1.9 (1.4-2.7, 1-16.6)	1.9 (1.4-2.7, 1-8.6)
Location, n (%)	Right	90 (35.3%)	58 (31.5%)
	Left	146 (57.3%)	112 (60.9%)
	Bilateral	19 (7.5%)	14 (7.6%)
Index image type, n (%)	CT with contrast	172 (67.5%)	135 (73.4%)
	CT without contrast	31 (12.2%)	27 (14.7%)
	CT with and without contrast	23 (9.0%)	14 (7.6%)
	MRI, any protocol	24 (9.4%)	8 (4.3%)
	FDG-PET	5 (2.0%)	0 (0%)
CT density features*^b^* (n = 246)	Total unenhanced CT	79 (31.0%)	59 (32.1%)
	HU unenhanced, median (IQR, range)	9 (5-31, −20-68)	9 (4-26, −20-68)
	HU unenhanced <10	40/79 (50.6%)	32/59 (54.2%)
	HU unenhanced ≥10	39/79 (49.4%)	27/59 (45.8%)
	Total adrenal protocol CT	31 (12.2%)	19 (10.3%)
	Absolute HU washout,*^c^* median (IQR)	60.5 (39.2-71.0)	52 (39.2-68.8)
	Absolute washout >60%	16/31 (51.6%)	8/19 (42.1%)
	Relative HU washout,*^c^* median (IQR)	45.7 (18.2-61.9)	44.8 (18.2-62.2)
	Relative washout >40%	20/31 (64.5%)	12/19 (63.2%)
CT imaging features (n = 246)	Calcifications	9 (3.7%)	4 (2.2%)
	Macroscopic fat	8 (3.2%)	3 (1.6%)
	Cystic change	8 (3.2%)	0 (0%)
	Hemorrhagic	8 (3.2%)	4 (2.2%)
MRI imaging features (n = 57)	Signal loss present	19 (33.3%)	14 (24.6%)
Radiology report for index image	Impression mentions mass	195 (76.4%)	135 (73.4%)
	Recommends follow-up imaging	99 (38.9%)	83 (45.1%)
	Recommends hormonal evaluation	7 (2.7%)	4 (2.2%)

Continuous data are summarized as mean and SD or as median and IQRs, unless otherwise indicated. Categorical data are presented as frequencies and percentages. Data are present for all individuals unless otherwise noted in table line.

Abbreviations: CT, computed tomography; FDG-PET, fluorodeoxyglucose–positron emission tomography; HU, Hounsfield units; IQR, interquartile range; MRI, magnetic resonance imaging;

^*a*^Incidentaloma defined as greater than 1-cm adrenal mass detected on imaging that was obtained for evaluation of pathology unrelated to adrenal disease.

^*b*^CT density characteristics reviewed from any preindex, index, or postindex imaging available.

^*c*^Absolute washout defined as [early postintravenous phase HU − delayed phase HU]/[early postintravenous phase HU − noncontrast HU] × 100]. Relative washout defined as [early postintravenous phase HU − delayed phase HU]/[early postintravenous phase HU] × 100].

**Figure 3:**
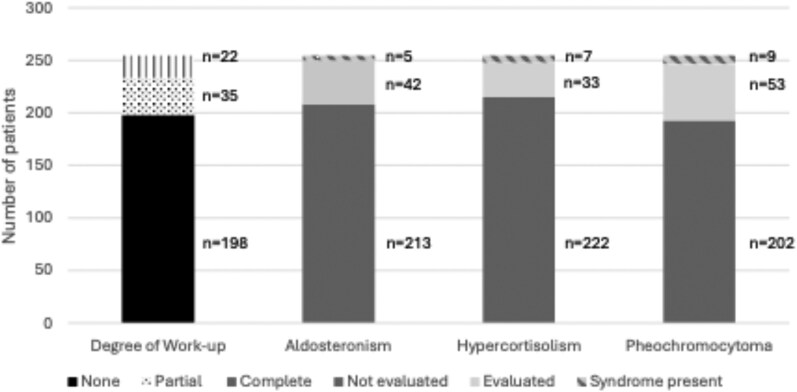
Hormone evaluation. Extent of hormone evaluation in all patients with adrenal masses, and prevalence of clinical disease.

Fig. 1 summarizes final diagnoses based on all available clinical, biochemical, radiologic, and pathologic data, or deemed indeterminate if key elements of evaluation were not available. Most adrenal masses lacked any hormonal evaluation (198, 78%) and very few had complete hormonal evaluation based on patient comorbidities (22, 8.6%) (Figs. 1 and 3; Supplementary Table S2 [16]). Aldosteronism screening was required for complete evaluation in patients with underlying hypertension (n = 89) and completed in 42 of 89 patients (47.2%). Pheochromocytoma screening was required for complete evaluation in patients with indeterminate or lipid-poor adrenal masses (n = 215) and completed in 40 of 215 (18.6%); 13 additional patients with lipid-rich masses also had screening, resulting in a total of 53 patients who had pheochromocytoma screening in the cohort. In total, adrenal masses with hormonal excess including CS, MACS, hyperandrogenism, and hyperaldosteronism accounted for 5.1% [13] and pheochromocytomas accounted for 3.5% [9]. Among those tested, the proportion of those with disease was high: hypercortisolism (7/29, 24%), hyperaldosteronism (5/42, 11%), and pheochromocytoma (9/53, 17%) (see Figs. 1 and 3). An endocrinologist or endocrine surgeon was involved in close to a fourth of cases (58, 23%) (see Table 1), and those seen at a specialty clinic were more likely to have biochemical evaluation (81.0% vs 5.1%; *P* < .001) (see Supplementary Table S2 [16]).

Among all adrenal masses, prevalence of malignancy was 16% (41), with 90% (37) of those being metastases to the adrenal (see Fig. 1), most commonly from breast and musculoskeletal malignancies (Supplementary Table S3 [16]). Univariable analysis for predictors of malignancy and pheochromocytoma identified nonincidental mechanism of discovery (OR 14.3; 95% CI, 6.9-29.7; *P* < .001), index size at detection (per 1-cm increase) (OR 1.5; 95% CI, 1.2-1.9; *P* < .001), change in lipid content (per each 10-HU increase) (OR 2.2; 95% CI, 1.4-4.1; *P* < .001), and male sex (OR 1.9; 95% CI, 1.01-3.8; *P* = .048) (Table 3); age was not a statistically significant predictor of malignancy. In a multivariable analysis of age, mode of discovery, tumor size, and sex, nonincidental mechanism of discovery (OR 15.5; 95% CI, 7.2-35.7; *P* < .0001) and index size at detection (per 1-cm increase) (OR 1.5; 95% CI, 1.2-2.0; *P* < .001) were associated with malignancy, whereas male sex was not (OR 2.0; 95% CI, 0.9-4.6; *P* = .08). Subgroup analysis by age demonstrated higher median density masses among individuals younger than 35 years (18 HU vs 7.5 HU on unenhanced CT; *P* = .05); however, there were no statistically significant differences in malignancy diagnoses between the groups (Table 4).

**Table 3:** Predictors of malignancy and pheochromocytoma, univariable and multivariable analyses (n = 255)

	Frequency (vs nonmalignancy/pheochromocytoma)	Univariable analysis, OR (95% CI), *P*	Multivariable analysis, OR (95% CI), *P*
Age (each 1-y decrease), median	34.5 (IQR 28.8-38, range 19-41) vs 36 (IQR 31-39, range 19-41)	1.04 (0.99-1.10), *P* = .13	0.97 (0.9-1.04), *P* = .35
Sex, male	16 (32%) vs 55 (47.3%)	1.9 (1.01-3.8), *P* = .048	2.0 (0.9-4.6), *P* = .08
Mechanism of detection, nonincidental	37 (74.0%) vs 34 (16.6%)	14.3 (6.9-29.7), *P* < .001	15.5 (7.2-35.7), *P* < .0001
Index size (each 1-cm increase), median	2.5 cm (IQR 1.7-3.7, range 1-16.6) vs 1.8 (IQR 1.4-2.6, range 1-9.5)	1.5 (1.2-1.9), *P* < .001	1.5 (1.2-2.0), *P* < .001
HU unenhanced*^a^* (each 10-HU increase), median	39.5 (IQR 29-44.5, range 29-46) vs 8 (IQR 3-26, range −20-68)	2.2 (1.4-4.1), *P* < .001	NA; unable to include in multivariable analysis due to this information being available for only 79 of 255 patients

Continuous data are summarized as mean and SD or as median and IQRs, unless otherwise indicated. Categorical data are presented as frequencies and percentages. Data are present for all individuals unless otherwise noted in table line. Logistic regression models were used to estimate the association between each predictor and malignancy, and results were reported as ORs with 95% CIs and corresponding *P* values, with values less than .05 considered statistically significant.

Abbreviations: HU, Hounsfield units; IQR, interquartile range; OR, odds ratio.

^*a*^Unenhanced HU were available for 79 patients.

**Table 4:** Subgroup analysis by age (n = 255)

Category	Subcategory	Age ≤35 y (n = 131)	Age >35 y (n = 124)	*P*
Age, median (IQR), y		31 (27 to 33)	39 (37 to 40)	N/A
Sex	Female	77 (58.7%)	65 (52.4%)	.31
Race	White	87 (68.0%)	69 (57.0%)	.07
Tobacco use, current or past		59 (51.3%)	65 (56.5%)	.42
Genetic predisposition syndrome present		15 (11.4%)	1 (0.8%)	<.001
Charlson comorbidity index		0 (0 to 2)	0 (0 to 2)	.97
Medical history	BMI (median, IQR)	29.4 (24.4 to 37.2)	29.9 (25.9 to 38.4)	.75
	Hypertension present	31 (23.6%)	58 (46.8%)	<.001
	Diabetes or prediabetes present	13 (9.9%)	25 (20.2%)	.02
	Hyperlipidemia present	9 (6.9%)	17 (13.7%)	.07
Mechanism of discovery	Incidental finding	93 (70.1%)	91 (73.4%)	.67
Imaging features	HU, median (IQR, range) (n = 79)*^a^*	18 (6 to 32 to −18 to 68)	7.5 (0.5 to 21.3 to −20 to 61)	.05
	HU < 10	17 (40.5%)	23 (60.5%)	.09
	Size, median (IQR, range)	2.2 (1.5 to 3.1 to 1 to 16.6)	1.8 (1.4 to 2.6, 1 to 9.5)	.24
Radiology reporting	Benign features	25 (35.7%)	26 (40.6%)	.56
Final diagnosis	Overt hormone excess	8 (6.1%)	8 (6.5%)	.91
	Adrenal metastases	21 (16.0%)	16 (12.9%)	.32
	ACC	3 (2.3%)	1 (0.8%)	.62
	Pheochromocytoma	7 (5.3%)	2 (1.6%)	.17

Continuous data are summarized as mean and SD or as median and IQRs, unless otherwise indicated. Categorical data are presented as frequencies and percentages. Data are present for all individuals unless otherwise noted in table line. Comparisons between groups were made with Pearson chi-square test for categorical data and Mann-Whitney for continuous data, due to nonnormal distribution of data.

Abbreviations: ACC, adrenocortical carcinoma; BMI, body mass index; CT, computed tomography; HU, Hounsfield units; IQR, interquartile range; N/A, not available.

^*a*^n = 79, available noncontrasted CT for determination of HU.

### Adrenal incidentalomas subgroup analysis

Among adrenal incidentalomas (184/255, 72%), prevalence of malignancy (10/184, 5.4%) or pheochromocytoma (3/184, 1.6%) was lower when compared to the cohort as a whole (Table 5). Malignancy breakdown included adrenal metastasis (9/184, 4.9%) and adrenocortical carcinoma (ACC) (1/184, 0.5%). Clinical characteristics associated with increased risk of malignancy or pheochromocytoma among adrenal incidentalomas included younger patient age (33 vs 36 years; *P* = .04), presence of genetic predisposition syndrome (15.4% vs 1.2%; *P* = .03), lower BMI (24.4 vs 30.7, *P* = .045), and larger tumor size at discovery (3.5 cm vs 1.8 cm; *P* < .001) (see Table 5).

**Table 5:** Patient characteristics associated with malignancy or pheochromocytoma among adrenal incidentalomas (n = 184)

	All incidentalomas (n = 184)	Malignancy or Pheochromocytoma*^a^* (n = 13)	Nonmalignancy or indeterminate diagnosis (n = 171)	*P*
Age, median (IQR), y	35.5 (31-39)	33 (24.5-36)	36 (31-39)	.04
Female sex, No. (%)	99 (53.8%)	9 (69.2%)	90 (52.6%)	.25
Smoking, past or present, No. (%)	95 (56.9%)	5 (38.5%)	90 (58.4%)	.16
Genetic predisposition syndrome, present,*^b^* No. (%)	4 (2.2%)	2 (15.4%)	2 (1.2%)	.03
BMI, median (IQR)	30.4 (25.8 to 38.4)	24.4 (20.7 to 36.1)	30.7 (26.5 to 38.7)	.045
Hypertension, present, n (%)	66 (35.9%)	5 (38.5%)	61 (35.7%)	.84
Diabetes, present, n (%)	33 (17.9%)	0 (0%)	33 (19.3%)	.12
Hyperlipidemia, present, n (%)	17 (9.2%)	0 (0%)	17 (9.9%)	.61
Tumor size, median (IQR, range), cm	1.9 (1.4 to 2.7 to 1 to 8.6)	3.5 (2.4 to 6.2 to 1 to 8.2)	1.8 (1.4 to 2.6 to 1 to 8.6)	<.001
HU, median (IQR, range)	9 (4 to 26, −20 to 68) n = 59*^c^*	38.5 (31-46, 31-46) n = 2	8 (3.5 to 24.5 to −20 to 68) n = 57	.08

Continuous data are summarized as mean and SD or as median and IQRs, unless otherwise indicated. Categorical data are presented as frequencies and percentages. Comparisons between groups were made with Pearson chi-square test for categorical data and Mann-Whitney for continuous data, due to nonnormal distribution of data.

Abbreviations: BMI, body mass index; CT, computed tomography; HU, Hounsfield units; IQR, interquartile range.

^*a*^Group included adrenocortical carcinoma (n = 1), metastasis (n = 9), and pheochromocytoma (n = 3).

^*b*^Incidental findings among genetically predisposed individuals were defined based on indication for imaging; for example, if adrenal mass was detected due to cross-sectional imaging for nephrolithiasis, this was considered incidental detection.

^*c*^n = 59, available noncontrasted CT for determination of HU

## Discussion

In this retrospective cohort study evaluating the characteristics of adrenal masses in young adults, we found a high frequency of malignancy and metabolic disease burden with obesity, hypertension, and diabetes. Less than 10% of young adults with adrenal masses had comorbidity-appropriate biochemical evaluation; yet among those evaluated, 20% had biochemical evidence of hypercortisolism and 3.5% had pheochromocytomas. Our findings highlight the need for comprehensive adrenal mass evaluation in young adults.

### Current landscape of adrenal mass characterization in young adults

Guidelines vary with regard to recommendations for monitoring and surgical indications for adrenal masses [3, 6-8, 18, 19]. Among large-society guidelines, 3 [3, 7, 18] recommend expedited evaluation in individuals younger than 40 years due to increased risk of malignancy or hormone activity; however, these are nongraded best practice statements due to the limited data in this subpopulation. To this point, some studies found a higher malignancy risk or hormone activity in adrenal masses in young adults [20, 21], while others contest this [22].

These discordant observations may be partially accounted for by low representation of young adults in study cohorts, which is likely due to decreased prevalence of adrenal masses and decreased acquisition of abdominal imaging in this age group [23]. On review of larger population-based or radiologic studies, it is difficult to determine the exact number of young adults who have been studied due to lack of clear specifications [4, 5, 9, 10, 24-26]. Several authors of these previous studies kindly shared unpublished data regarding this demographic, which is highlighted in Table 6. Among studies in which age cohort data are available, young adults represented between 7% and 18% of the total study cohort [4, 5, 9, 10].

**Table 6:** Summary of studies characterizing adrenal masses

Author, y (study design)	Study design and population characteristics	Total cohort size and key findings participants with adrenal mass	Total young adult*^a^* participants and key findings in young adults
Jacob, 2025 (current study) (retrospective cohort)	Tertiary referral center St Louis, MO, USA Consecutive adults with clinical evaluation for adrenal masses, 72% adrenal incidentaloma; 78% did not have any hormone work-up	N/A	Age 18-40 y: 255 Median age 35 y (IQR 30-39 y) Malignancy: 16.1% (ACC 1.6%, adrenal metastasis 14.5%) Pheochromocytoma: 3.5% Hormone hypersecretion: 5.1%; (hyperaldosteronism 2% and cortisol autonomy 2.7%). Nonfunctional adenomas 3.9%.
Hamidi,*^b^* 2024 [10] (retrospective cohort)	Tertiary referral center Rochester, MN, USA Consecutive adults with clinical evaluation for incidental adrenal adenoma (all nonadenomas were excluded); 15% did not have complete hormone work-up).	N = 1516; median age 59 y (IQR 18-91 y) Malignancy: N/A (excluded from study) Pheochromocytoma: N/A (excluded from study) Hormone hypersecretion: 38% (hyperaldosteronism 4% and cortisol autonomy 34%). Nonfunctional adenomas 46%.	Age 18-40 y: n = 275 Malignancy: N/A (excluded from study) Pheochromocytoma: N/A (excluded from study) Hormone hypersecretion: not separately reported for age subgroup 18-40 y
Nasiroğlu, 2024 [25] (retrospective cohort)	Tertiary referral center Ankara, Turkey Adults with clinical evaluation of adrenal incidentalomas. Complete hormone evaluation was required for inclusion.	N = 431; mean age 55 (SD 11 y) Malignancy: not reported Pheochromocytoma: 3.9% Hormone hypersecretion: 23.4% (hyperaldosteronism 3.9% and cortisol autonomy 15.6%). Nonfunctional adenomas 76.6%	Age 18-40 y: not specified Malignancy, pheochromocytoma, and hormone hypersecretion: not separately reported for age subgroup 18-40 y
Suntornlohanakul,*^b^* 2024 [9] (retrospective cohort)	Tertiary referral center Birmingham, UK Consecutive adults with clinical evaluation for adrenal masses, > 60% incidentalomas; 14.5% did not have complete hormone work-up.	N = 1397; median age 60 y (IQR 49-70 y) Malignancy: 16.3% (ACC 10.6%, adrenal metastases 5.7%) Pheochromocytoma: 12.7% Hormone excess: 30% (hyperaldosteronism 5.2% and cortisol autonomy 25.2%). Nonfunctional adenomas 37.7%.	18-40 years: 192 Malignancy, pheochromocytoma, and hormone hypersecretion not separately reported for age subgroup 18-40 y Authors report younger median age (44 y) in those with Cushing syndrome
Jing, 2022 (cross sectional) [5]	Community health examination center Chongqing, China Elective abdominal imaging for detection of adrenal incidentalomas, with subsequent clinical evaluation; 37% did not have complete hormone work-up.	n = 351; mean age 56 y (SD 12 y) Malignancy: 0% Pheochromocytoma: 0% Hormone hypersecretion: 30.7% (hyperaldosteronism 11.8% and cortisol autonomy 18.9%).	Age 18-45 y: 52 Malignancy: 0% Pheochromocytoma: 0% Hormone hypersecretion: present in 27.8% of young adults who underwent screening; authors report no differences in hormone secretion by age
Ebbehoj, 2020 [4] (retrospective population-based cohort)	Population based study Olmstead County, MN, USA Patients diagnosed with adrenal mass with subsequent clinical evaluation; > 80% adrenal incidentalomas; 85% did not have complete hormone work-up.	n = 1247; median age 62 y (range 0-96 y) Malignancy: 8.6% Pheochromocytoma: 8.6% Hormone hypersecretion: 4.1% (hyperaldosteronism 3.7% and cortisol autonomy 0.4%).	Age 18-40 y: 92 Malignancy: 4.3% Pheochromocytoma: 5.4% Hormone hypersecretion: not separately reported for age subgroup 18-40 y
Ichijo, 2020 [24] (cross-sectional)	1014 hospitals across Japan Consecutive adults with clinical evaluation for adrenal incidentalomas	n = 3672; mean age 58 y (SD 13 y) Malignancy: 5.2% (ACC 1.4%, adrenal metastasis 3.8%) Pheochromocytoma: 8.5% Hormone hypersecretion: 15.6% (hyperaldosteronism 5.1% and cortisol autonomy 10.5%). Nonfunctional adenoma 50.8%	18-40 years: not specified Malignancy, pheochromocytoma, and hormone hypersecretion: not separately reported for age subgroup 18-40 y
Yilmaz, 2020 [26] (retrospective cohort)	Tertiary referral center Antalya, Turkey Consecutive adults with clinical evaluation for adrenal incidentalomas. Complete hormone evaluation was required for inclusion.	n = 755; median age 56 y (range, 18-86 y) Malignancy: 3% (ACC 1.5%, adrenal metastasis 1.5%) Pheochromocytoma: 3.8% Hormone hypersecretion: 24.4% (hyperaldosteronism 3.7% and cortisol autonomy 20.7%). Nonfunctional adenomas 71.8%.	Age 18-40 y: not specified Malignancy, pheochromocytoma, and hormone hypersecretion: not separately reported for age subgroup 18-40 y
**Total No. and characteristics of young adult participants*^a^***			866 Malignancy*^c^*: 4.3-16.1% Pheochromocytoma*^c^*: 3.5-5.4% Hormone hypersecretion*^c^*: 5.1-27.8%

Abbreviations: ACC, adrenocortical carcinoma; N/A, not available.

^*a*^Defined as age 18 to 40 years or 18 to 45 years as per study protocol.

^*b*^The authors wish to express their sincere gratitude to the researchers who kindly provided supplemental, unpublished data regarding the number of young adults in their respective cohorts.

^*c*^Only studies that described subgroup analysis by age were used to determine these ranges.

### Prevalence of malignancy

Past studies demonstrate considerable variability in estimates of malignancy among adrenal masses, ranging between 3% and 16.3% [4, 9, 24, 26]. This range likely reflects differences in study design and inclusion criteria, where clinical cohorts are enriched with more suspicious adrenal masses, and radiologic cohorts may be more reflective of true prevalence, but limited by incomplete evaluations. Most studies, however, do not report malignancy prevalence by age.

In one study that approximated malignancy risk by age group, Ebbehoj and colleagues [4] reported 4.3% malignancy among the 92 young adults within their population-based cohort of 1287 in Olmstead County, Minnesota, compared with 8.6% among the population as a whole. In our cohort of 255 adrenal masses in young adults, prevalence of malignancy in the entire cohort was 16%, which surpasses rates demonstrated in other studies [4, 5, 24]. Notably, variations in methodology limit direct comparisons between these studies and ours when discussing overall prevalence of malignancy and hormone functionality. The majority of these malignancies were due to metastatic disease; interestingly, while renal cell carcinoma and lung adenocarcinoma are classically the most common to metastasize to the adrenal [27], breast and musculoskeletal primary cancers were the most common in our younger cohort. Possible explanations may be related to referral patterns in our oncologic center, as well as the relative prevalence of breast and musculoskeletal malignancies compared with other primary malignancies in this age group.

Our findings are concordant with prior studies suggesting that diagnosis of malignancy is more common in masses found on imaging ordered for cancer staging or hormone excess evaluation compared to those masses found incidentally. Higher overall malignancy diagnoses in our cohort compared to prior studies may reflect a true higher prevalence rate among young adults or may be reflective of a selection bias from the study being conducted at a tertiary oncologic referral center. There may also be an imaging selection bias indicating that young adults who have more comorbidities are more likely to have imaging, selecting for a sicker patient cohort.

In analysis of all patients with pheochromocytoma or malignancy regardless of mechanism of discovery, nonincidental mechanism of discovery and larger size at discovery were found to be predictive both in univariable and multivariable regression models, which supports findings from prior studies [4, 9, 24]. Male sex was associated with higher risk for these outcomes in univariable models but lost statistical significance in multivariable models, and we were unable to include unenhanced HU due to limited numbers of patients within the cohort for whom these data were available; further studies may be needed to better understand the effects of these clinical factors. The percentage of tumors harboring malignancy was similar by age, though we found a significantly higher frequency of lipid-poor masses among patients younger than 35 years; this group may benefit from particular attention in malignancy assessments of identified adrenal masses.

### Prevalence of hormone excess syndrome

Most of our cohort was not screened for hormonal excess; however, among the patients who had complete hormonal evaluation, prevalence of hormone excess was high. Other studies used a clinical cohort of patients with an adrenal mass that required complete hormonal evaluation for inclusion in the study; among these, hormone hypersecretion was approximately 24%, without available subgroup analysis by age [9, 10, 25, 26]. Another study performed subgroup analysis in a young adult population (ages 18-45 years) with hormone hypersecretion reported at 27.8% [5]; however, 37% of patients were missing hormone evaluation, which limits interpretation.

Individuals in our cohort of young adults with adrenal masses had multiple metabolic comorbidities, which parallels or exceeds prevalence in population-based studies [28-30]. Prior studies have suggested increased risks of metabolic disease in those with adrenal masses [31-35], possibly related to glucocorticoid autonomy. In our study, cortisol after 1-mg DST was abnormal in 41% of those tested (9/22; see Supplementary Table S2 [16]). Multiple analyses have shown that patients with abnormal cortisol suppression testing had higher rates of new-onset or progressive diabetes and hypertension, and increased cardiovascular events [11, 31, 36]. Nonfunctional adenomas have also been associated with higher all-cause and cardiovascular mortality [31, 33], and several small studies have shown higher rates of resistant hypertension and insulin resistance in those with nonfunctioning adenomas compared to those without adrenal adenomas [32, 34, 35]. It is difficult to assess if the high rates of metabolic disease in our cohort are related to the presence of adrenal masses or other factors. Nevertheless, since cardiovascular and metabolic risk associated with untreated MACS rises over time, we would argue that younger patients with MACS may have the most to gain from prompt diagnosis and treatment of adrenal hormone hypersecretion [11, 37].

### Factors influencing evaluation

Our study demonstrates that patients are more likely to have appropriate evaluation and follow-up if seen by endocrinologists or endocrine surgeons, likely due to greater awareness of disease management in these subspecialities. In our cohort, less than a quarter had evaluation with specialists, but within this group, 81% had a biochemical work-up compared to the 5.1% who had an evaluation who were not seen by specialists. Prior studies also suggest wording of radiology reports and their impressions may effectively influence clinical behavior and increase rates of evaluation [38-40]. Radiology imaging reports recommended follow-up imaging in 38.9% and biochemical evaluation in only 2.7% (see Table 2). These factors may explain the low radiologic and biochemical evaluation rates in our cohort.

Variations in recommendations and management may be explained by evolving and occasionally conflicting recommendations between radiology, endocrinology, and endocrine surgery guidelines. For example, while both American College of Radiology (ACR) and European Network for the Study of Adrenal Tumors (ENSAT) guidelines recommend no further imaging for lesions that are lipid rich (HU <10) [3, 8], the American Association of Clinical Endocrinologists recommends repeat imaging for up to 2 years [19]. ENSAT and ACR differ, however, in recommendations for indeterminant masses smaller than 4 cm in size, with ENSAT recommending immediate additional imaging for characterization and ACR recommending this only if the mass is larger than 2 cm in size [3, 8].

Biochemical assessment recommendations also vary by groups. For example, the Korean Endocrine Society suggests evaluation for pheochromocytoma in all adrenal masses, but ENSAT and Canadian Urologic Association recommend it only in patients with lipid-poor masses [3, 7, 18]. Biochemical evaluation recommendations in radiology guidelines have also shifted in the last decade: The 2017 ACR white paper on the management of adrenal incidentalomas recommends only “consideration” of biochemical evaluation for adrenal incidentalomas, but updates in 2019 recommend biochemical evaluation in all masses, which aligns with other major guidelines [3, 6-8]. It is possible that this shift, which occurred during the years that our study was conducted, is an explanation for the low rates of radiology recommendations for biochemical screening in our cohort. ACR guidelines understandably focus on radiologic parameters associated with malignancy, which have high immediate importance on detection of an adrenal mass; nonetheless, biochemical abnormalities are common and radiology reporting practices could play an important role in increasing clinician awareness of necessary biochemical evaluation.

### Limitations and strengths

The observational and retrospective nature of this study has inherent limitations, and selection biases may have resulted from being conducted in a tertiary referral center. Radiologic cohort development may be affected by missed cases of adrenal masses related to heterogeneous reporting terminology (eg, adrenal cysts and hematomas may have been missed with our search strategy); nonetheless, our goal was to comprehensively capture all detected adrenal masses for malignancy risk and hormonal evaluation, and therefore adrenal pathology unrelated to this goal may have been underrepresented. It is also possible that we missed follow-up imaging if patients used other health systems in the region. With regard to the hormonal evaluation, biochemical evaluation was incomplete for most adrenal masses, which mirrors findings of other studies [4], but limits classification for final diagnosis or the frequency of hormonal excess among this patient population. Additionally, frequency of malignancy or hormonal excess among tested individuals may be contributed to by selection bias, with biochemical testing being conducted in patients with more severe disease presentations. As a result, we cannot reliably conclude that young adults have a higher prevalence of malignancy or hormone hypersecretion compared to other age groups.

Nonetheless, a major strength of our study is the large sample size, as well as systematic acquisition of index cases through a radiology database. We attempted to optimize data reliability through secondary imaging review by abdominal radiologists and secondary clinical data review by the study team. Another strength is a greater diversity of our population, with 39% of individuals being from racial or ethnic minority backgrounds.

Additional areas of research may include studying the hormonal status of adrenal masses in young adults in a multicenter cohort to overcome issues of selection bias and increase the rates of those with complete hormonal evaluation.

### Conclusion

In our cohort of 255 young adults with adrenal masses, we found a 16% prevalence of malignancy overall, and a 5% prevalence of malignancy in patients with adrenal incidentalomas. Factors associated with the diagnoses of malignancy and pheochromocytoma were nonincidental mechanism of discovery and larger size at time of detection. We found low rates of biochemical screening for hormone excess despite nearly half of patients having at least one metabolic complication. Our study emphasizes the need for education of the clinical community regarding appropriate evaluation of adrenal masses, and further studies to clarify how patient age may affect clinical outcomes of adrenal masses.

## Data Availability

Some or all datasets generated during and/or analyzed during the current study are not publicly available but are available from the corresponding author on reasonable request.

## Abbreviations

ACC: adrenocortical carcinoma
ACR: American College of Radiology
BMI: body mass index
CS: Cushing syndrome
CT: computed tomography
DST: dexamethasone suppression test
ENSAT: European Network for the Study of Adrenal Tumors
FAP: familial adenomatous polyposis
FDG-PET: fluorodeoxyglucose–positron emission tomography
HU: Hounsfield units
IQR: interquartile range
LCMS: liquid chromatography–tandem mass spectrometry
MACS: mild autonomous cortisol secretion
MEN1: multiple endocrine neoplasia 1
MEN2: multiple endocrine neoplasia 2
MRI: magnetic resonance imaging
NF1: neurofibromatosis type 1
NF2: neurofibromatosis type 2
OR: odds ratio
VHL: von Hippel-Lindau

## References

[1] Song JH, Chaudhry FS, Mayo-Smith WW. The incidental adrenal mass on CT: prevalence of adrenal disease in 1,049 consecutive adrenal masses in patients with no known malignancy. AJR Am J Roentgenol. 2008;190(5):1163‐1168. 18430826 10.2214/AJR.07.2799

[2] Reimondo G, Castellano E, Grosso M, et al Adrenal incidentalomas are tied to increased risk of diabetes: findings from a prospective study. J Clin Endocrinol Metab. 2020;105(4):e973‐e981. 10.1210/clinem/dgz284 31900474

[3] Fassnacht M, Tsagarakis S, Terzolo M, et al European Society of Endocrinology clinical practice guidelines on the management of adrenal incidentalomas, in collaboration with the European Network for the Study of Adrenal Tumors. Eur J Endocrinol. 2023;189(1):G1‐G42. 37318239 10.1093/ejendo/lvad066

[4] Ebbehoj A, Li D, Kaur RJ, et al Epidemiology of adrenal tumours in Olmsted County, Minnesota, USA: a population-based cohort study. Lancet Diabetes Endocrinol. 2020;8(11):894‐902. 33065059 10.1016/S2213-8587(20)30314-4 PMC7601441

[5] Jing Y, Hu J, Luo R, et al Prevalence and characteristics of adrenal tumors in an unselected screening population: a cross-sectional study. Ann Intern Med. 2022;175(10):1383‐1391. 36095315 10.7326/M22-1619

[6] Yip L, Duh QY, Wachtel H, et al American association of endocrine surgeons guidelines for adrenalectomy: executive summary. JAMA Surg. 2022;157(10):870‐877. 35976622 10.1001/jamasurg.2022.3544 PMC9386598

[7] Rowe NE, Kumar RM, Schieda N, et al Canadian Urological Association guideline: diagnosis, management, and followup of the incidentally discovered adrenal mass. Can Urol Assoc J. 2023;17(2):12‐24. 36849113 10.5489/cuaj.8248 PMC9970641

[8] Glazer DI, Mayo-Smith WW. Management of incidental adrenal masses: an update. Abdom Radiol. 2020;45(4):892‐900. 10.1007/s00261-019-02149-2 31359097

[9] Suntornlohanakul O, Mandal S, Saha P, et al Presentation and management of patients with adrenal masses: a large tertiary centre experience. Eur J Endocrinol. 2024;191(5):481‐490. 39425921 10.1093/ejendo/lvae131

[10] Hamidi O, Shah M, Zhang CD, et al Clinical and imaging presentations are associated with function in incidental adrenocortical adenomas: a retrospective cohort study. Eur J Endocrinol. 2024;191(1):47‐54. 38941271 10.1093/ejendo/lvae078 PMC11234193

[11] Deutschbein T, Reimondo G, Di Dalmazi G, et al Age-dependent and sex-dependent disparity in mortality in patients with adrenal incidentalomas and autonomous cortisol secretion: an international, retrospective, cohort study. Lancet Diabetes Endocrinol. 2022;10(7):499‐508. 35533704 10.1016/S2213-8587(22)00100-0 PMC9679334

[12] Kirsch MJ, Hsu KT, Lee MH, et al Hormonal evaluation of incidental adrenal masses: the exception, not the rule. World J Surg. 2020;44(11):3778‐3785. 32651604 10.1007/s00268-020-05679-9

[13] Schumm M, Hu MY, Sant V, et al Automated extraction of incidental adrenal nodules from electronic health records. Surgery. 2023;173(1):52‐58. 36207197 10.1016/j.surg.2022.07.028

[14] Davenport E, Lang Ping Nam P, Wilson M, Reid A, Aspinall S. Adrenal incidentalomas: management in British district general hospitals. Postgrad Med J. 2014;90(1065):365‐369. 24686243 10.1136/postgradmedj-2013-132386

[15] Feeney T, Talutis S, Janeway M, et al Evaluation of incidental adrenal masses at a tertiary referral and trauma center. Surgery. 2020;167(5):868‐875. 31672517 10.1016/j.surg.2019.07.034

[16] Jacob S, Genere N. Characterization of adrenal masses in young adults at a tertiary care academic medical center. Figshare. 2025. Published online November 1, 2025. 10.6084/m9.figshare.29982892.v1 PMC13071808 41982342

[17] Charlson ME, Pompei P, Ales KL, MacKenzie CR. A new method of classifying prognostic comorbidity in longitudinal studies: development and validation. J Chronic Dis. 1987;40(5):373‐383. 3558716 10.1016/0021-9681(87)90171-8

[18] Lee JM, Kim MK, Ko SH, et al Clinical guidelines for the management of adrenal incidentaloma. Endocrinol Metab. 2017;32(2):200‐218. 10.3803/EnM.2017.32.2.200 PMC5503865 28685511

[19] Zeiger MA, Thompson GB, Duh QY, et al American Association of Clinical Endocrinologists and American Association of Endocrine Surgeons medical guidelines for the management of adrenal incidentalomas. Endocr Pract. 2009;15:1‐20. 10.4158/EP.15.S1.1 19632967

[20] Foo E, Turner R, Wang KC, et al Predicting malignancy in adrenal incidentaloma and evaluation of a novel risk stratification algorithm. ANZ J Surg. 2018;88(3):E173‐E177. 28118677 10.1111/ans.13868

[21] Mantero F, Terzolo M, Arnaldi G, et al A survey on adrenal incidentaloma in Italy. Study group on adrenal tumors of the Italian Society of Endocrinology. J Clin Endocrinol Metab. 2000;85(2):637‐644. 10690869 10.1210/jcem.85.2.6372

[22] Iñiguez-Ariza NM, Kohlenberg JD, Delivanis DA, et al Clinical, biochemical, and radiological characteristics of a single-center retrospective cohort of 705 large adrenal tumors. Mayo Clin Proc Innov Qual Outcomes. 2018;2(1):30‐39. 30225430 10.1016/j.mayocpiqo.2017.11.002 PMC6124341

[23] Smith-Bindman R, Chu PW, Azman Firdaus H, et al Projected lifetime cancer risks from current computed tomography imaging. JAMA Intern Med. 2025;185(6):710‐719. 40227719 10.1001/jamainternmed.2025.0505 PMC11997853

[24] Ichijo T, Ueshiba H, Nawata H, Yanase T. A nationwide survey of adrenal incidentalomas in Japan: the first report of clinical and epidemiological features. Endocr J. 2020;67(2):141‐152. 31694993 10.1507/endocrj.EJ18-0486

[25] Nasiroğlu Imga N, Aslan Y, Çatak M, Aykanat İC, Tuncel A, Berker D. Clinical, radiological, and surgical outcomes of 431 patients with adrenal incidentalomas: retrospective study of a 10-year single-center experience. Turk J Med Sci. 2024;54(2):376‐383. 39050392 10.55730/1300-0144.5802 PMC11265901

[26] Yilmaz N, Avsar E, Tazegul G, Sari R, Altunbas H, Balci MK. Clinical characteristics and follow-up results of adrenal incidentaloma. Exp Clin Endocrinol Diabetes. 2021;129(5):349‐356. 31958848 10.1055/a-1079-4915

[27] Panwar V, Cai Q, Jia L. Metastatic diseases to the adrenal gland: a comprehensive study from an academic institution with emphasis on clinical occult cases. Pathol Res Pract. 2024;261:155487. 39079382 10.1016/j.prp.2024.155487

[28] Hales CM, Carroll MD, Fryar CD, Ogden CL. Prevalence of obesity and severe obesity among adults: United States, 2017–2018. National Center for Health Statistics Data Brief 360. Centers for Disease Control and Prevention; 2020; (360):1-8. Accessed September 11, 2023. https://www.niddk.nih.gov/health-information/health-statistics/overweight-obesity

[29] Ostchega Y, Fryar CD, Nwankwo T, Nguyen DT. Hypertension prevalence among adults aged 18 and over: United States, 2017–2018. National Center for Health Statistics Data Brief 364. Centers for Disease Control and Prevention; 2020; (364):1-8. Accessed September 11, 2023. https://www.cdc.gov/nchs/products/databriefs/db364.htm

[30] Gwira JA, Fryar CD, Gu Q. Prevalence of total, diagnosed, and undiagnosed diabetes in adults: United States, August 2021–August 2023. National Center for Health Statistics Data Brief 516; 2024; (516). Accessed September 11, 2023. https://www.cdc.gov/nchs/products/databriefs/db516.htm 10.15620/cdc/165794 PMC12035661 40085919

[31] Dalmazi GD, Vicennati V, Garelli S, et al Cardiovascular events and mortality in patients with adrenal incidentalomas that are either non-secreting or associated with intermediate phenotype or subclinical Cushing's syndrome: a 15-year retrospective study. Lancet Diabetes Endocrinol. 2014;2(5):396‐405. 24795253 10.1016/S2213-8587(13)70211-0

[32] Kim JH, Kim MJ, Lee JH, Yoon JW, Shin CS. Nonfunctioning adrenal incidentalomas are not clinically silent: a longitudinal cohort study. Endocr Pract. 2020;26(12):1406‐1415. 33471732 10.4158/EP-2020-0182

[33] Patrova J, Mannheimer B, Lindh JD, Falhammar H. Mortality in patients with nonfunctional adrenal tumors. JAMA Intern Med. 2023;183(8):832‐838. 37358871 10.1001/jamainternmed.2023.2442 PMC10294015

[34] Arruda M, Mello Ribeiro Cavalari E, Pessoa de Paula M, et al The presence of nonfunctioning adrenal incidentalomas increases arterial hypertension frequency and severity, and is associated with cortisol levels after dexamethasone suppression test. J Hum Hypertens. 2018;32(1):3‐11. 10.1038/s41371-017-0011-4 29176595

[35] de Paula MP, Moraes AB, de Souza MDGC, et al Cortisol level after dexamethasone suppression test in patients with non-functioning adrenal incidentaloma is positively associated with the duration of reactive hyperemia response on microvascular bed. J Endocrinol Invest. 2021;44(3):609‐619. 32686043 10.1007/s40618-020-01360-z

[36] Morelli V, Reimondo G, Giordano R, et al Long-term follow-up in adrenal incidentalomas: an Italian multicenter study. J Clin Endocrinol Metab. 2014;99(3):827‐834. 24423350 10.1210/jc.2013-3527

[37] Kjellbom A, Lindgren O, Danielsson M, Olsen H, Löndahl M. Mortality not increased in patients with nonfunctional adrenal adenomas: a matched cohort study. J Clin Endocrinol Metab. 2023;108(8):e536‐e541. 36800277 10.1210/clinem/dgad074 PMC10348456

[38] Eldeiry LS, Alfisher MM, Callahan CF, Hanna NN, Garber JR. The impact of an adrenal incidentaloma algorithm on the evaluation of adrenal nodules. J Clin Transl Endocrinol. 2018;13:39‐45. 29998066 10.1016/j.jcte.2018.07.001 PMC6037878

[39] de Haan RR, Schreuder MJ, Pons E, Visser JJ. Adrenal incidentaloma and adherence to international guidelines for workup based on a retrospective review of the type of language used in the radiology report. J Am Coll Radiol. 2019;16(1):50‐55. 30253931 10.1016/j.jacr.2018.08.011

[40] Corwin MT, Arora A, Loehfelm TW, Fananapazir G, Campbell MJ. Adherence to guidelines for hormonal evaluation in patients with incidentally detected adrenal nodules: effects of radiology report wording and standardized reporting. Abdom Radiol N Y. 2020;45(9):2910‐2915. 10.1007/s00261-020-02517-3 32270262

